# Molecular analysis suggests that Namibian cheetahs (*Acinonyx jubatus*) are definitive hosts of a so far undescribed *Besnoitia* species

**DOI:** 10.1186/s13071-021-04697-3

**Published:** 2021-04-14

**Authors:** Gereon Schares, Maike Joeres, Franziska Rachel, Mareen Tuschy, Gábor Á. Czirják, Pavlo Maksimov, Franz J. Conraths, Bettina Wachter

**Affiliations:** 1 Institute of Epidemiology, Friedrich-Loeffler-Institut, Federal Research Institute for Animal Health, Südufer 10, 17493 Greifswald-Insel Riems, Germany; 2 Department of Wildlife Diseases, Leibniz Institute for Zoo and Wildlife Research, Alfred-Kowalke-Street 17, 10315 Berlin, Germany; 3 Department of Evolutionary Ecology, Leibniz Institute for Zoo and Wildlife Research, Alfred-Kowalke-Street 17, 10315 Berlin, Germany

**Keywords:** *Besnoitia* spp., Namibia, Real-time PCR, Metatheria, Eutheria, Placental mammals, Marsupial mammals, Phylogeny, *Acinonyx jubatus*

## Abstract

**Background:**

*Besnoitia darlingi*,* B. neotomofelis* and *B. oryctofelisi* are closely related coccidian parasites with felids as definitive hosts. These parasites use a variety of animal species as intermediate hosts. North American opossums (*Didelphis virginiana*), North American southern plains woodrats (*Neotoma micropus*) and South American domestic rabbits (*Oryctolagus cuniculus*) are intermediate hosts of *B. darlingi*, *B. neotomofelis* and *B. oryctofelisi*, respectively. Based on conserved regions in the internal transcribed spacer-1 (ITS1) sequence of the ribosomal DNA (rDNA), a real-time PCR for a sensitive detection of these *Besnoitia* spp. in tissues of intermediate hosts and faeces of definitive hosts has recently been established. Available sequence data suggest that species such as *B. akodoni* and *B. jellisoni* are also covered by this real-time PCR. It has been hypothesised that additional *Besnoitia* spp. exist worldwide that are closely related to *B. darlingi* or *B. darlingi*-like parasites (*B. neotomofelis*, *B. oryctofelisi*, *B. akodoni* or *B. jellisoni*). Also related, but not as closely, is *B. besnoiti*, the cause of bovine besnoitiosis.

**Methods:**

Faecal samples from two free-ranging cheetahs (*Acinonyx jubatus*) from Namibia that had previously tested positive for coccidian parasites by coproscopy were used for this study. A conventional PCR verified the presence of coccidian parasite DNA. To clarify the identity of these coccidia, the faecal DNA samples were further characterised by species-specific PCRs and Sanger sequencing.

**Results:**

One of the samples tested positive for *B. darlingi* or *B. darlingi*-like parasites by real-time PCR, while no other coccidian parasites, including *Toxoplasma gondii*, *Hammondia hammondi*, *H. heydorni*,* B. besnoiti* and *Neospora caninum*, were detected in the two samples. The rDNA of the *B. darlingi*-like parasite was amplified and partially sequenced. Comparison with existing sequences in GenBank revealed a close relationship to other *Besnoitia* spp., but also showed clear divergences.

**Conclusions:**

Our results suggest that a so far unknown *Besnoitia* species exists in Namibian wildlife, which is closely related to *B. darlingi*, *B. neotomofelis*,* B. oryctofelisi*,* B. akodoni *or *B. jellisoni.* The cheetah appears to be the definitive host of this newly discovered parasite, while prey species of the cheetah may act as intermediate hosts.

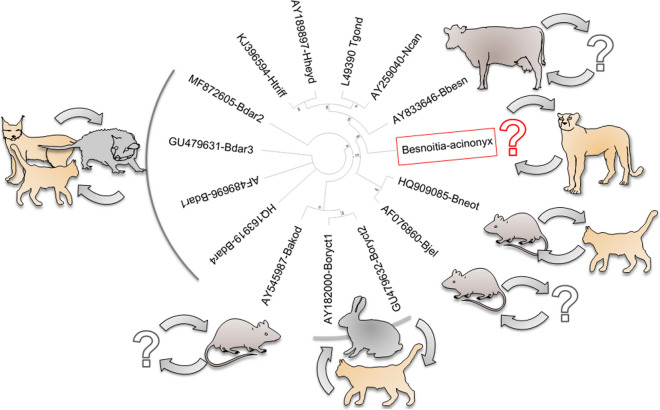

**Supplementary Information:**

The online version contains supplementary material available at 10.1186/s13071-021-04697-3.

## Background

*Besnoitia darlingi*, *B. neotomofelis* and *B. oryctofelisi* are closely related coccidian parasites, for which domestic cats have been ascertained as definitive hosts [1–4]. The bobcat (*Lynx rufus*) has been identified as the definitive host of *B. darlingi* in the wild [5]. *Besnoitia darlingi* uses a marsupial, the North American opossum (*Didelphis virginiana*), as its intermediate host [5]. In contrast, *B. neotomofelis* and *B. oryctofelisi* have been described in placental mammals, i.e. in the North American southern plains woodrat (*Neotoma micropus*) and in domestic rabbits from South America (*Oryctolagus cuniculus*), respectively [2, 4, 6]. *Besnoitia akodoni*, another closely related *Besnoitia* species, was described in a placental mammal in South America, i.e. the rodent *Akodon montensis*, as intermediate host [7]. Another *Besnoitia* sp., *B. jellisoni*, was described in the North American white-footed deer mouse (*Peromyscus maniculatus*) and in three species of kangaroo rats (*Dipodomys* species) as intermediate hosts [8, 9]. In contrast to *B. darlingi*, *B. neotomofelis*, and *B. oryctofelisi*, the definitive hosts of *B. jellisoni* or *B. akodoni* are unknown. Domestic cats, other carnivorous mammals, various birds and snakes have been excluded as final hosts of *B. jellisoni* [10, 11]. Further reports suggest the presence of similar *Besnoitia* spp. parasites in New Zealand, Australia, Japan and Kenya [12–15]. Moreover, for *B. wallacei*, first described on Oahu, Hawaii, in a domestic cat (i.e. its definitive host), experimental studies suggested rodents (mice, rats) as appropriate intermediate hosts [10].

It has therefore recently been argued that other, so far unknown, *Besnoitia* species may exist in other parts of the world [16]. The high level of conservation in the internal transcribed spacer-1 (ITS1) sequence of the ribosomal DNA (rDNA) among related species of *B. darlingi* and *B. darlingi*-like species (*B. neotomofelis*,* B. oryctofelisi*,* B. akodoni*, *B. jellisoni*) was utilised to establish primers and a probe for the detection of such *Besnoitia* spp. parasites in their intermediate (e.g. rodents, lagomorphs and marsupials) or definitive hosts (e.g. wild felids or canids) [16]. This real-time PCR is not able to detect the *Besnoitia* spp. of ungulates, i.e. cattle, goats, donkeys and horses, and caribou and reindeer, such as *B. besnoiti*, *B. caprae*, *B. bennetti* and *B. tarandi*, respectively [16].

Our previous work suggested the presence of *Toxoplasma gondii* in most of 12 Namibian wildlife species and that of *B. besnoiti* and *Neospora caninum* in a few of these same species, including six of suborder Feliformia, four of suborder Caniformia and two of suborder Ruminantia [17]. Felids, including cheetahs (*Acinonyx jubatus*), are known as definitive hosts of *T. gondii*, but for *B. besnoiti*, the causative agent of bovine besnoitiosis, the definitive host is still unknown, although wild felids have been discussed as candidates [17, 18]. Since morphological identification of coccidian parasites is challenging, we used molecular methods to examine the faeces collected from the ampulla recti of two free-ranging cheetahs for *B. besnoiti.* In a previous study, these two cheetahs had been shown to be positive for coccidian oocysts by coproscopy [19].

## Methods

### DNA extraction

In this study, we used faecal samples from two free-ranging female cheetahs, one sub-adult and one adult, from farmland in central Namibia. The animals had previously tested positive for coccidian parasites by coproscopy [19]. The original and detailed records reported up to 3600 oocysts with an approximate size of 18–22.5 × 18.0–36.0 µm per gram faeces [19]. Coproscopy of the sub-adult and the adult cheetah for the present study revealed 3600 and 50 oocysts with an approximate size of 18.0 × 18.0 µm, respectively, per gram faeces, as well as 300 oocysts with an approximate size of 22.5 × 36.0 µm per gram faeces in the adult cheetah (BW and GÁC, unpublished data). Capture and handling of the animals, sample collection, transport and storage has been described previously [17, 19, 20]. The Quick-DNA Fecal/Soil Microbe DNA Miniprep Kit (Zymo Research Europe GmbH, Freiburg, Germany) was used to extract DNA from approximately 200-mg aliquots according to the manufacturer’s recommendations. From the faecal sample of the sub-adult female, two aliquots were available, which were independently extracted. Extraction typically yields 100 μl DNA per faecal sample [16].

### Endpoint PCR

To test for coccidian parasites, a PCR was performed using the common apicomplexan small subunit ribosomal DNA (*18S* rDNA) primers COC-1 and COC-2 [21, 22]. *Hammondia heydorni* DNA was tested using the primers JS4 and JS5 as described [23, 24]. Due to the high level of sequence identity in the rDNA target, the primer pair JS4/JS5 was expected to amplify also DNA of *Hammondia triffitae*, a coccidian parasite using foxes as definitive hosts [25–27].

**Fig. 1:**
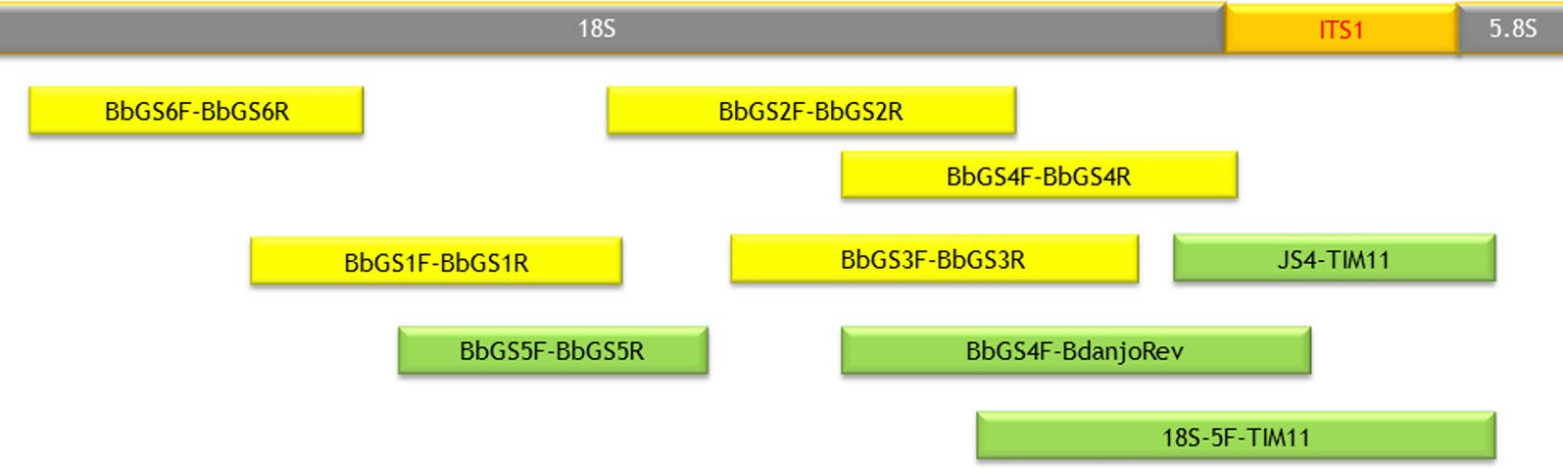
Overview of PCR fragments and primer names used to assess rDNA sequences of *Besnoitia darlingi*-like parasites (named “Besnoitia-acinonyx” for this study) and related organisms in faeces of a Namibian cheetah. Green-coloured amplicons revealed sequences closely related to *B. darlingi*-like parasites (Table 1); yellow-coloured amplicons revealed sequences related to additional coccidia (Table 1). Details on primer sequences are given in Additional file 1: Table S1.* ITS1* Internal transcribed spacer-1

For the identification of coccidian parasites by Sanger sequencing, rDNA was amplified by endpoint PCR using primer pairs (Fig. 1) as previously published [23, 28, 29] and listed in Additional file 1: Table S1.

For all PCRs, primers were used at a final concentration of 0.5 mM and dNTPs at a final concentration of 250 mM each (Stratec Molecular GmbH, Berlin, Germany). Taq polymerase (Stratec Molecular GmbH) had a final concentration of 1 U/25 µl using the buffer system supplied with the enzyme. The PCR cycling conditions were: 94 °C, 5 min; then 56 °C/1 min (with 0.5 °C decrement per cycle after the 1st cycle), 72 °C/1 min, 94 °C/1 min, for 10 cycles; followed by 51 °C/1 min, 72 °C/1 min, 94 °C/1 min, for 40 cycles; and a final incubation at 51 °C for 1 min and a final extension at 72 °C for 5 min.

### Real-time PCRs

Real-time PCRs were used to test for *T. gondii*, *Hammondia hammondi*, *B. besnoiti*,* B. darlingi* and *B. darlingi*-like parasites or *N. caninum*. *Toxoplasma gondii* was examined as previously reported targeting the TgREP-529 repetitive element [30, 31]. *Hammondia hammondi* was diagnosed using a recently published real-time PCR targeting the HhamREP-529 repetitive element [22]. In the case of *B. besnoiti*, a fragment of the ITS1 region in the rDNA was amplified as described (BbRT1; [32]). For *B. darlingi* and *B. darlingi*-like parasite, a recently published real-time PCR designated BdanjoRT1 was applied [16]. For the detection of *N. caninum* DNA, a previously published real-time PCR targeting the Nc5 gene [31, 33] was used.

To monitor the inhibition of the real-time PCRs, a heterologous plasmid with DNA sequences resembling the enhanced green fluorescent protein (EGFP) gene [34] was added to the reaction mix in all real-time PCRs except those for *N. caninum*. The internal control PCR included the primers EGFP1-F, EGFP2-R and the probe EGFP1 [22]. A 712-bp fragment of the EGFP gene was amplified and cloned into the pGEM-Teasy standard cloning vector (Promega, Walldorf, Germany) in reverse orientation to obtain the internal control (IC) DNA (pGEM-EGFP2-rev). The amount of the IC DNA added to each reaction was adjusted so that it resulted in a quantification cycle (Cq) value of approximately 32 in the real-time PCR.

Reactions were performed in a final volume of 20 µl using a commercial master mix (PerfeCTa MultiPlex qPCR ToughMix; Quantabio, VWR International, Darmstadt, Germany) and a CFX384 instrument (Biorad Laboratories GmbH, Munich, Germany). Primers and probes were purchased from MWG-Biotech (Ebersberg, Germany). Standard concentrations for primers (500 nM) and probes (100 nM, target specific primers; 160 nM, EGFP1) were used. The cycling conditions in real-time PCR: were 95.0 °C, 5 min (initial denaturation); then 95.0 °C/10 s, 60.0 °C/30 s, for 45 cycles. After each cycle, the light emission by the fluorophore was measured. Real-time PCR results were analysed using the CFX manager software version 1.6 (Biorad Laboratories GmbH, Munich, Germany).

### Cloning

For Sanger sequencing of the amplification products, bands of the expected size were excised from agarose gels and purified with a commercial kit (NucleoSpin® Gel and PCR Clean-up; Macherey–Nagel, Düren, Germany), following the manufacturer’s instructions. Purified amplification products were then cloned into a commercially available vector (pGEM®-T Easy Vector System I; Promega, Mannheim, Germany) and used to transform chemically competent *Escherichia coli* (OneShot TOP10; Thermo Fisher Scientific, Langenselbold, Germany). The transformed *E. coli* cell were cultivated and the plasmid DNA was subsequently collected using a commercial kit (QIAprep Spin Miniprep Kit; Qiagen, Hilden, Germany) according to the manufacturer’s instructions. Sequencing was performed using the BigDye Terminator v1.1 Cycle Seq. Kit (Thermo Fisher Scientific) and passage through NucleoSEQ Columns (Macherey–Nagel) for cleaning up the nucleic acids, in an ABI 3130 capillary sequencer (Thermo Fisher Scientific).

The forward and reverse sequences were aligned, if necessary trimmed based on primer sequence information, and the consensus sequences for the individual cloned amplification products compared to sequences stored in GenBank, EMBL, DDBJ or RefSeq using BLASTn with standard conditions.

### Phylogenetic analysis

The evolutionary history based on ITS1 rDNA sequences was inferred using the maximum parsimony (MP) method. The number of base substitutions per site between sequences included into the analysis was termed “pairwise distance” or “evolutionary divergence” in the following section. The MP tree was obtained using the subtree-pruning-regrafting algorithm [35] with search level 0, in which the initial trees were obtained by the random addition of sequences (10 replicates). All codon positions (1st, 2nd, 3rd, noncoding) were included. Evolutionary analyses were conducted in MEGA X [36].

## Results

### PCRs for coccidian parasites

The observed parasitic structures, previously diagnosed microscopically as coccidia [19], were confirmed by a positive reaction in an* 18S* rDNA-based endpoint COC-1/COC-2 PCR for both samples of the two cheetahs (one sub-adult and one adult female). Species-specific PCRs were negative for *T. gondii*, *H. hammondi* and *B. besnoiti*. The unlikely presence of *N. caninum* or *H. heydorni* was also excluded by real-time or endpoint PCR, respectively. Using a real-time PCR established to detect *B. darlingi* and *B. darlingi*-like parasites [16], we observed a positive signal (Cq 28.3 or 31.9) in both sample aliquots of the sub-adult female cheetah. This suggests that genomes equivalent to 10–100 tachyzoites were present in 10 µl of the 100 µl of DNA extracted from this faecal sample. No signal was observed in the sample of the adult female cheetah, although the IC real-time PCR revealed no inhibition.

### Characterisation of *18S* and ITS1 rDNA sequences of a *Besnoitia* sp.-like parasite

The positive *B. darlingi*-like real-time PCR suggested either the presence of *B. darlingi* in the faeces of the positive cheetah—although this was unlikely because *B. darlingi* use marsupials as intermediate hosts, which are not present in Africa—or, most likely, the presence of oocysts of another, possibly not yet known, *Besnoitia* species. To identify this parasite, we partially characterised its rDNA using overlapping amplification products for parts of the *18S* rDNA, the ITS1 rDNA and parts of the *5.8S* rDNA (Fig. 1; Table 1; Additional file 1: Table S1). The sequences of the cloned amplicons were analysed by BLASTn with recording of the five top species hits using the BLASTn suite (Max Score). The amplicons of four different targets, using the primer pairs JS4–TIM11, 18S-5F–TIM11, BbGS4F–BdanjoRev and BbGS5F–BbGS5R, revealed sequences related to the candidate of a *Besnoitia* species (Table 1). These were *B. darlingi*, *B. neotomofelis*, *B. oryctofelisi*, *B. jellisoni*, *B. akodoni*, *B. besnoiti*, *B. bennetti*,* B. caprae* and *B. tarandi*, with percent identities ranging up to 99.54% among the top species hits in BLASTn (Table 1; *Besnoitia* sp.-related). With the exception of the BbGS4F-BdanjoRev-cloned sequences*, B. darlingi* sequences (GenBank, EMBL, DDBJ, RefSeq) always ranked first (Table 1), which suggests that a parasite closely related to *B. darlingi* or *B. darlingi*-like parasites had been excreted as oocysts by the Namibian cheetah. The sequences of the JS4–TIM11 (*n* = 6), 18S-5F–TIM11 (*n* = 3) and BbGS4F–BdanjoRev (*n* = 3) clones, i.e. clones covering the ITS1 sequence, were aligned, and the consensus sequence was stored at GenBank (MW468050) using the parasite isolate designation “Besnoitia-acinonyx”. The *B. darlingi*-like sequence amplified by BbGS5F–BbGS5R (i.e. a part of the *18S* rDNA) was also stored in GenBank (MW559556).

**Table 1:** The PCR analyses targeted nine overlapping regions of the *18S* rDNA, internal transcribed spacer-1 rDNA and part of the *5.8S* rDNA

Relatedness of sequences	Amplification with primer pairs	Number of clones	Top five species with highest identity and coverage in GenBank (number of sequences per organism, percent coverage, percent identity)
*Besnoitia* sp.-related	BbGS4F–BdanjoRev	3	*Besnoitia oryctofelisi* (1, 99–100, 98.58–99.01), *B. darlingi* (1, 99–100, 98.31–98.73), *B. tarandi* (1, 100, 97.85–98.17), *B. besnoiti* (7, 99–100, 97.85–98.17), *B. bennetti* (1, 99–100, 97.69–98.03)
	JS4–TIM11	3	*Besnoitia darlingi* (3, 82–90, 93.60–94.20), *B. oryctofelisi* (2, 77–80, 93.42–93.91), *B. caprae* (1, 100, 87.02–88.03), *B. besnoiti* (5, 100, 87.02–88.06), *B. akodoni* (1, 76–77, 92.52–92.97)
	18S-5F–TIM11	3	*Besnoitia darlingi* (1, 92, 95.75–96.02), *B. oryctofelisi* (1, 89–90, 95.93–96.19), *B. besnoiti* (7, 99–100, 91.41–91.82), *B. tarandi* (1, 96–97, 91.32–91.72), *B. bennetti* (1, 93, 91.04–91.46)
	JS4–TIM11	2	*Besnoitia darlingi* (3, 79–90, 92.86–93.40), *B. oryctofelisi* (2, 75–78, 92.73–92.97), *B. caprae* (1, 100, 86.44–86.81), *B. besnoiti* (5, 100, 86.44–86.81), *B. bennetti* (1, 100, 86.21–86.60)
	JS4–TIM11	1	*Besnoitia darlingi* (2, 80–89, 91.95–92.82), *B. oryctofelisi* (2, 85–86, 92.69–92.82), *B. akodoni* (1, 83, 91.72), *B. neotomofelis* (1, 83, 90.72), *B. bennetti* (1, 100, 85.54)
	BbGS5F–BbGS5R	2	*Besnoitia darlingi* (2, 99–100, 99.31–99.54), *B. oryctofelisi* (1, 99–100, 99.31–99.54), *B. jellisoni* (1, 99–100, 99.31–99.54), *B. besnoiti* (12, 100, 99.09–99.31), *B. tarandi* (2, 100, 98.86–99.31)
Coccidia-related	BbGS1F–BbGS1R	6	*Cystoisospora* sp. ex. *Aonyx cinereus* (1, 99, 99.17–99.67), *C. belli* (8, 63, 99.17–99.67), *C. ohioensis* (4, 63–99, 99.00–99.50), *C. suis* (2, 63–99, 99.00–99.50), *Cystoisospora* sp. (1, 63–99, 99.00–99.50)
	BbGS4F–BbGS4R	2	*Cystoisospora belli* (5, 100, 99.66), *C. timori* (1, 100, 99.66), *C.* cf. *ohioensis* (1, 100, 99.49), *H. triffitae* (1, 100, 99.33), *H. heydorni* (1, 100, 99.33)
	BbGS6F–BbGS6R	2	*Cystoisospora ohioensis* (4, 97–100, 99.76–99.53), *C. belli* (5, 100, 99.30–99.53), *C. timori* (1, 100, 99.06), *C. canis* (2, 97, 99.76), *C. suis* (2, 97, 99.76)
	BbGS2F–BbGS2R	1	*Cystoisospora* sp. ex. *Aonyx cinereus* (1, 100, 100.00), *C. canis* (2, 100, 100.00), *C. ohioensis* (3, 100, 100.00), *C. suis* (2, 100, 100.00), *C. laidlawi* (1, 100, 100.00)
	BbGS3F–BbGS3R	1	*B. besnoiti* (4, 100, 99.81), *B. darlingi* (1, 100, 99.81), *T. gondii* (19, 100, 99.81), *H. heydorni* (1, 100, 99.81), *H. hammondi* (1, 100, 99.81)
	BbGS3F–BbGS3R	1	*Cystoisospora ohioensis* (4, 100, 99.81), *C. suis* (2, 100, 99.81), *C. belli* (2, 100, 99.81), *Cystoisospora* sp. (1, 100, 99.81), *C. timori* (1, 100, 99.81)
Unrelated to coccidia	BbGS6F–BbGS6R	1	*Thecaphora spilanthes* (1, 70, 93.64), uncultured basidiomycete (1, 67, 92.89), *Exobasidium rhododentri* (1, 70, 90.36), uncultured Ceriporiopsis (1, 72, 89.44), *Exobasidium rostrupli* (1, 70, 90.07),
	BbGS6F–BbGS6R	1	*Thecaphora spilanthes* (1, 45, 98.79), *Arabidopsis lyrate* (1, 56, 99.09), *Scyliorhinus canicula* (1, 34, 99.09), uncultured archeon (1, 40, 99.08), uncultured bacterium (4, 40, 99.08)
	BbGS4F–BbGS4R	1	*Helminthosporium hispanicum* (2, 100, 89.92), *H. tiliae* (2, 100, 89.92), *H. quercium* (2, 100, 89.92), *H. austriacum* (2, 100, 89.92), *H. velutium* (3, 100, 89.92)
	BbGS4F–BbGS4R	1	*Thecaphora saponariae* (1, 75, 99.65), *Thecaphora amaranthi* (1, 76, 90.12), *Exobasidiomycetes* sp. (1, 78, 89.09), *Tilletiopsis washingtonensis* (1, 78, 88.76), *Tilletiopsis* sp. (1, 78, 88.76)

A variable number of plasmid clones per target was received and subsequently Sanger-sequenced. Sequences were not concatenated, but individually assessed by BLASTn. The top five hits of species using in BLASTn the option MaxScore are displayed. Due to the conserved primer regions, sequences of unrelated species, i.e. predominantly fungi, were identified in addition to sequences resembling sequences of coccidian parasite-related or *Besnoitia* sp.-related species

Some of the remaining sequences had only coccidian parasites among the first five species hits. However, these species hits were dominated by *Cystoisospora* spp., which may suggest that *Cystoisospora* spp. had been present in the faecal samples in addition to the *B. darlingi*-like parasites (Table 1;  Coccidia-related).

### Phylogenetic relationships to “Besnoitia-acinonyx”

**Fig. 2:**
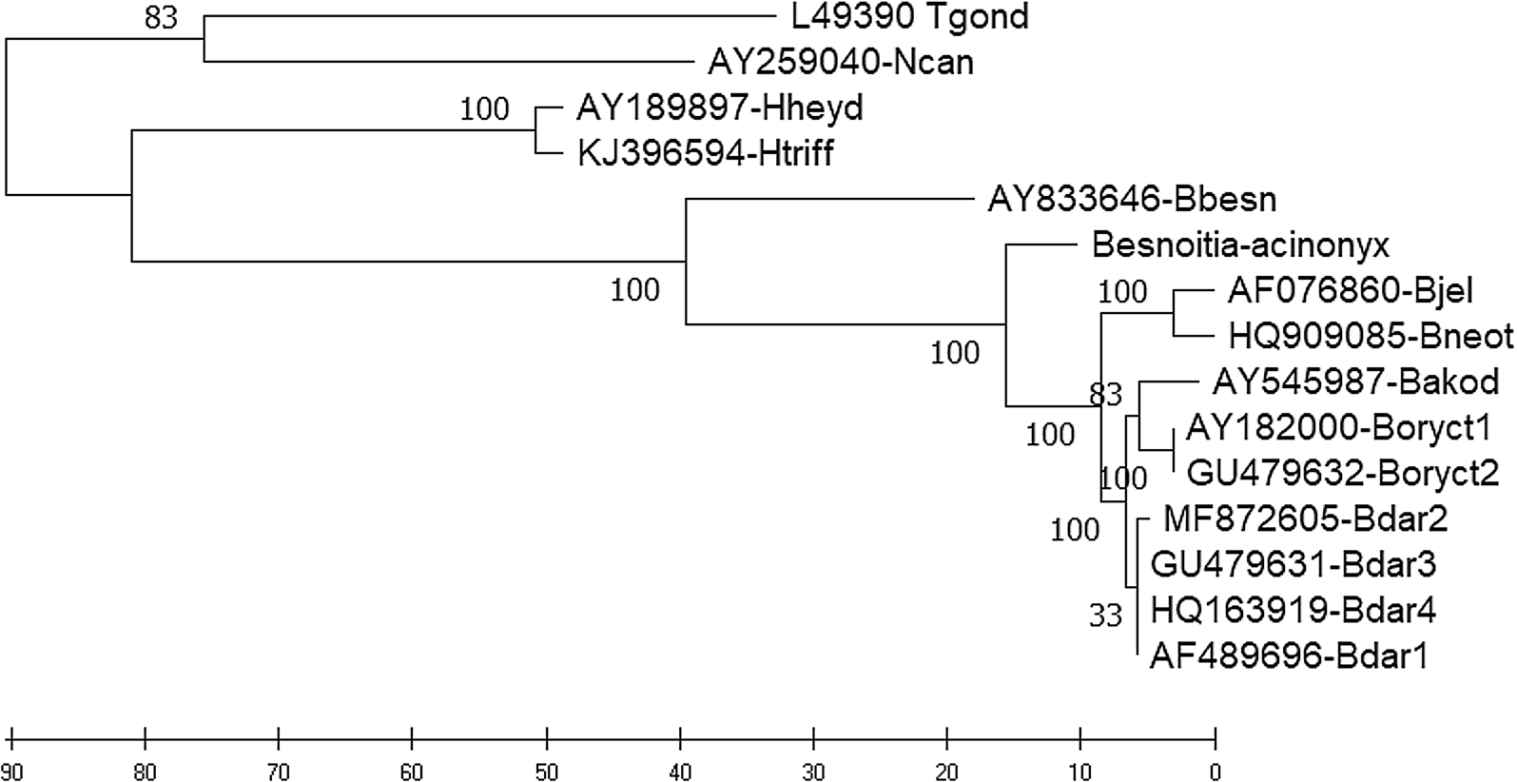
GenBank sequences of *Neospora caninum* (*Ncan*; AY259040), *Toxoplasma gondii* (*Tgond*; ME49, L49390), *Hammondia heydorni* (*Hheyd*; AY189897), *Hammondia triffitae* (*Htriff*; KJ396594), *Besnoitia besnoiti* (*Bbesn*, AY833646), “Besnoitia-acinonyx” (MW468050; this study; Additional file 2: Text S1), *B. jellisoni* (*Bjel*; AF076860), *B. neotomofelis* (*Bneot*; HQ909085), *B. akodoni* (*Bakod*; AY545987), *B. oryctofelisi* #1 (*Boryct1*; AY182000), *B. oryctofelisi* #2 (*Boryct2*; GU479632), *B. darlingi* #1 (*Bdar1*; AF489696), *B. darlingi* #2 (*Bdar2*; MF872605), *B. darlingi* #3 (*Bdar3*; GU479631) and *B. darlingi* #4 (*Bdar4*, HQ163919) were subjected to evolutionary history analysis using the maximum parsimony (MP) method. The consensus tree inferred from the 6 most parsimonious trees is shown. Branches corresponding to partitions reproduced in < 50% trees are collapsed. The consistency index is 0.912 (0.866), the retention index is 0.910 (0.910), and the composite index is 0.830 (0.788) for all sites and parsimony-informative sites (in parentheses). The MP tree was obtained using the subtree-pruning-regrafting algorithm [35] with search level 0, in which the initial trees were obtained by the random addition of sequences (10 replicates). The tree is drawn to scale, with branch lengths calculated using the average pathway method [35] and are in the units of the number of changes over the whole sequence. The analysis involved 15 nucleotide sequences. All codon positions (1st, 2nd, 3rd, noncoding) were included. There were 425 positions in the final dataset. Evolutionary analyses were conducted in MEGA X [36]

Based on the ITS1 rDNA sequence, the possible phylogenetic relationships of the newly described species, represented by the DNA isolate (here termed “Besnoitia-acinonyx”), to other *Besnoitia* spp., namely *B. darlingi*, *B. neotomofelis*,* B. oryctofelisi*,* B. akodoni*,* B. jellisoni* and *B. besnoiti*, but also to *T. gondii*,* H. heydorni*,* H. triffitae* and *N. caninum*, were assessed. The ITS1 sequence placed “Besnoitia-acinonyx” between those of *B. darlingi*, *B. darlingi*-like parasites and *B. besnoiti* (Fig. 2). Estimates of evolutionary divergence revealed a close relationship to *B. darlingi*,* B. neotomofelis*, *B. oryctofelisi*,* B. akodoni* and *B. jellisoni* (pairwise distances < 0.1; Table 2) and a larger distance to *B. besnoiti* (distance 0.234; Table 2). Among the remaining coccidia tested, *T. gondii* showed a higher distance to “Besnoitia-acinonyx” (0.595; Table 2) than *N. caninum* (0.544; Table 2). Interestingly, *T. gondii* and *N. caninum* showed a closer relationship to “Besnoitia-acinonyx” than to all remaining *Besnoitia* spp. except *B. besnoiti* (Table 2). In addition, *B. besnoiti* was closer to “Besnoitia-acinonyx” (0.234; Table 2) than to any other *Besnoitia* sp. examined (0.294–0.268; Table 2). Identities of the ITS1 rDNA of “Besnoitia-acinonyx” with *B. darlingi* and the *B. darlingi*-like parasites were 89.7–90.1% (*B. darlingi*), 89.7% (*B. oryctofelisi*), 88.9% (*B. akodoni*) and 86.9% (*B. jellisoni* and *B. neotomofelis*).

**Table 2:** Estimates of evolutionary divergence between the internal transcribed spacer-1 rDNA region sequences of “Besnoitia-acinonyx” (MW468050; this study) and other *Besnoitia* spp. [*B. besnoiti* (AY833646), *B. jellisoni* (AF076860), *B. neotomofelis* (HQ909085), *B. akodoni* (AY545987), *B. oryctofelisi* (AY182000, GU479632), *B. darlingi* (AF489696, MF872605, GU479631, HQ163919], *Toxoplasma gondii* (L49390), *Neospora caninum* (AY259040), *Hammondia heydorni* (AY189897) and *H. triffitae* (KJ396594)

Isolate	Besnoitia-acinonyx	*T. gondii*	*N. caninum*	*H. heydorni*	*H. tiffitae*	*B. besnoiti*	*B. jellisoni*	*B. neotomofelis*	*B. akodoni*	*B. oryctofelisi* (*n* = 2 sequences)	*B. darlingi* (*n* = 4 sequences)
Besnoitia- acinonyx	*0.000*										
*Toxoplasma gondii*	0.595	*0.000*									
*Neospora caninum*	0.544	0.257	*0.000*								
*Hammondia heydorni*	0.542	0.288	0.269	*0.000*							
*H. triffitae*	0.531	0.297	0.268	0.011	*0.000*						
*Besnoitia besnoiti*	0.234	0.530	0.492	0.534	0.523	*0.000*					
*B. jellisoni*	*0.095*	0.674	0.674	0.608	0.596	0.279	*0.000*				
*B. neotomofelis*	*0.095*	0.692	0.657	0.610	0.598	0.288	*0.026*	*0.000*			
*B. akodoni*	*0.079*	0.617	0.628	0.570	0.558	0.294	*0.063*	*0.063*	*0.000*		
*B. oryctofelisi* (*n* = 2 sequences)^a^	*0.069*	0.639	0.609	0.540	0.529	0.268	*0.054*	*0.054*	*0.030*	*0.000*	
*B. darlingi* (*n* = 4 sequences)^b^	*0.064 *(*0.069*)	0.645 (0.654)	0.629 (0.638)	0.571 (0.578)	0.559 (0.567)	0.271 (0.277)	*0.049* (*0.053*)	*0.048* (*0.053*)	*0.026* (*0.030*)	*0.013* (*0.017*)	*0.000 *(*0.004*)

The number of base substitutions per site between sequences are shown in the table (pairwise distance). Analyses were conducted using the maximum composite likelihood model [37]. This analysis involved 15 nucleotide sequences. All codon positions (1st, 2nd, 3rd, Noncoding) were included. All ambiguous positions were removed for each sequence pair (pairwise deletion option). There were 425 positions in the final dataset. Evolutionary analyses were conducted in MEGA X [36]

Pairwise distances > 0.5 are underlined. Distances < 0.1 are given in italics

^a^The two sequences of *B. oryctofelisi* revealed the same values for all cells

^b^Three of the four sequences of *B. darlingi* revealed the same values for all cells; the fourth sequence of *B. darlingi* (MF872605) revealed slightly different values which are shown in parenthesis

## Discussion

In this study, we examined archived faecal samples of two free-ranging cheetahs from farmland in central Namibia. Coccidian parasites had been identified by coproscopy in these samples previously [19]. Using a coccidia-specific PCR, the microscopic observations were confirmed for both animals.

We originally expected *T. gondii* or *H. hammondi* in the cheetahs, as these parasites are known to use felids as definitive hosts [37]. Antibodies against the tachyzoite stage of *B. besnoiti* had been detected in blue wildebeest (*Connochaetes taurinus*) and lions (*Panthera leo*) in Namibia by serology [17]. Thus, in addition, we suspected that *B. besnoiti* might be present in faecal samples of Namibian cheetahs because felids might be a definitive (and/or intermediate) host of *B. besnoiti* [17, 18]. In southern Africa, the existence of *B. besnoiti*, which uses cattle as intermediate hosts, has long been known (summarised in [38]). *Besnoitia besnoiti*-like parasites have been previously isolated from or observed in prey animals of cheetah, such as blue wildebeest, impala (*Aepyceros melampus*) and kudu (*Tragelaphus strepsiceros*) in South Africa [39].

For reasons of completeness, DNA extracted from the faecal samples was also examined for *N. caninum* and *H. heydorni*, although these are parasites of dogs, dingoes, wolves or coyotes [27, 40], and for *B. darlingi*, which uses marsupials as intermediate hosts, as well as for *B. darlingi*-like parasites [16].

Using our previously developed real-time PCR, established to identify *B. darlingi*, *B. neotomofelis*,* B. oryctofelisi*,* B. akodoni* and *B. jellisoni* in intermediate and definitive hosts, we obtained positive results with two sample aliquots from one cheetah (Cq 28.3 and Cq 31.9). When this newly developed real-time PCR was first reported, we hypothesised that further *B. darlingi*-related parasites might exist worldwide and might be picked up by this PCR [16]. Since all *B. darlingi*-like parasites known so far have been detected in North or South America, we deemed it unlikely that the *B. darlingi*-like PCR signal that was observed in the faeces of the cheetah belonged to *B. darlingi* or one of the American *B. darlingi*-like parasites. Therefore, this study reports the first evidence for the existence of an additional *Besnoitia* sp. in southern Africa.

A part of the rDNA (*18S* rDNA) and in particular the ITS1 rDNA sequence of this parasite was characterised in more detail. In analogy to other coccidian parasites, such as *T. gondii*, we expected that the rDNA sequence would be present more than 100-fold in the genome of a single parasite organism [41], which makes the rDNA gene and particularly the ITS1 region a sensitive target for species identification. Since there were no purified oocysts from the faecal samples available, it was difficult to identify or amplify *Besnoitia* sp. DNA selectively from the plethora of organisms (most likely bacteria and fungi) present in the faecal samples.

Our real-time PCR results suggested that genomes equivalent to 10–100 tachyzoites were present in 10 µl of the 100 µl of DNA extracted from 200 mg of the positive faecal sample. This corresponds to about 60–600 up to 500–5000 oocysts per gram faeces, depending on the proportion of sporulated oocysts (assuming 8 genomes in sporulated oocyts and only a single genome in unsporulated oocysts from the gut). Since oocysts were collected from the ampulla recti—and were therefore unsporulated—the estimate of 500–5000 oocysts per gram of faeces seems more realistic. This estimation is in accord with the number of 3600 oocysts with an approximate size of 18 × 18 µm per gram faeces recorded in the previous coproscopy study [19]. However, oocysts of *B. darlingi*-like parasites have an expected size of 10 × 12 μm (*B. darlingi* [1, 5]), 11 × 12 µm (*B. oryctofelisi*, [1, 2]) or 13 × 14 μm (*B. neotomofelis*, [2]). Several scenarios are possible to explain this discrepancy. First, the oocysts of “Besnoitia-acinonyx” are 18 × 18 µm in size. Second, other coccidian parasites, probably *Cystoisospora* sp., were also present in the sample and oocysts with a diameter of 10–14 µm (expected for *Besnoitia* sp.) were overlooked. In addition to the oocyst sizes observed, the sequences of *18S* rDNA fragments amplified suggest that *Cystoisospora* sp. were also present in these faeces, which supports the second scenario. However, the observation of such sequences is not a final proof of the existence of *Cystoisospora* sp. because *18S* rDNA sequences are largely conserved and particular sequence fragments of the *18S* rDNA can belong to many different coccidian parasites [42]. Third, and a less likely scenario, it can be hypothesised that the observed “Besnoitia-acinonyx” DNA did not originate from oocysts, but from intermediate host stages in infected prey of the cheetah. Fourth, coprophagia as a source of the observed oocysts is very unlikely, as cheetahs display an extremely selective feeding behaviour and coprophagia has never been observed in this species [43]. Fifth, feeding from carcasses is also very rare in cheetahs [43]. Thus, future studies are necessary to isolate oocysts of this parasite from cheetahs to confirm that this species is the definitive host of “Besnoitia-acinonyx” and to determine the respective oocyst size of this *B. darlingi*-related parasite.

As several organisms (including other coccidia) may have been present in the faeces, we concentrated on sequences that belonged unambiguously to *B. darlingi*-like parasites. The BdanjoRev primer [16], which had been applied in the *B. darlingi* real-time PCR, played—in combination with the BbGS4F primer [28]—a central role in the identification of the correct *Besnoitia*-like rDNA sequences [16]. Using the previously published primer pair JS4 [23] and TIM11 [29], as well as the newly established primer 18S-5F in combination with TIM11, we observed exclusively *B. darlingi*-like sequences, which we aligned and made available as a provisional rDNA sequence of “Besnoitia-acinonyx” (MW468050).

The ITS1 region of the rDNA *Besnoitia* spp. of New World marsupials, rodents and domestic rabbits show only a few differences [5, 44]. The ITS1 rDNA sequence of the *Besnoitia* sp. observed in the Namibian cheetah was similar to previously described ITS1 rDNA sequences, but differed from all *B. darlingi* and *B. darlingi*-like sequences described to date. Identities of the ITS1 rDNA of “Besnoitia-acinonyx” with *B. darlingi* and *B. darlingi*-like parasites were ≤ 90% (i.e. 89.7–90.1% [*B. darlingi*], 89.7% [*B. oryctofelisi*], 88.9% [*B. akodoni*] and 86.9% [*B. jellisoni* and *B. neotomofelis*]). Compared among each other, *B. darlingi* and the remaining *B. darlingi*-like parasites (namely *B. oryctofelisi*, *B. akodoni*, *B. jellisoni* and *B. neotomofelis*), each characterised by different intermediate host spectra, showed much higher identities in the ITS1 rDNA region, ranging from 92.9% to 98.3%. Thus, it appears to be justified to conclude that the “Besnoitia-acinonyx” sequence belongs to a so far unknown *Besnoitia* sp. that most likely uses the cheetah as its definitive host. This view is supported further by identities among the ITS1 rDNAs of *Besnoitia* sp. of ungulates (namely *B. besnoiti*, *B. tarandi*, *B. bennetti*, *B. caprae*) which are almost 100% identical, but belong to clearly separate species.

Since no free-ranging marsupials exist in Africa, rodent or lagomorph species, which are prey for cheetahs, probably serve this parasite as intermediate hosts. In analogy to the South American *B. oryctofelisi*, lagomorphs, such as the Cape hare (*Lepus capensis*), the Savanna hare (*L. microtis*) or the Scrub hare (*L. saxatilis*), may represent suitable intermediate hosts.

The ITS1 rDNA sequence of “Besnoitia-acinonyx” suggests a closer relationship to the American *B. darlingi* and *B. darlingi*-like parasites than to *B. besnoiti*, which infects cattle and probably also antelopes in southern Africa. This finding suggests that all *B. darlingi* or *B. darlingi*-like parasites, regardless of their American or African origin and their ability to infect marsupials or placental mammals, have a common ancestor, which evolved when the South American and the African continents were not yet disconnected, i.e. 100–200 million years ago. Most likely, this common ancestor evolved together with marsupial and placental mammalian animals, which started to separate also around this time [45]. Marsupial mammals are the closest living relatives to placental mammals, sharing a common ancestor that lived approximaely 130 million years ago [45].

## Conclusion

Molecular analysis of a faecal sample revealed that Namibian cheetah (*Acinonyx jubatus*) is most likely a definitive host of a newly described *Besnoitia* species. This species is closely related to *B. darlingi* and other related *Besnoitia* spp. parasites of rodents and lagomorphs. Future studies are needed to identify its natural intermediate host in southern Africa, which most likely is a common prey of the Namibian cheetah. Hares, rabbits and rodents represent possible intermediate host candidates to be further examined.

## Supplementary Information

**Additional file 1: Table S1.** Primer sequences used to amplify rDNA in two aliquots of a faecal sample of a Namibian cheetah with the aim to characterise an unknown *Besnoitia* sp.

**Additional file 2: Text S1.** Sequence alignment to assemble the ITS1 rDNA and part of the *18S* and *5.8S* rDNAs of a *B. darlingi*-like parasite (here called “Besnoitia-acinonyx”) of Namibian cheetah (*Acinonyx jubatus*). The text file shows 12 amplicon-based sequences used to establish MW468050.

## Data Availability

Data supporting the conclusions of this article are included within the article and its additional files. The raw datasets used and analysed for the present study are available from the corresponding author upon reasonable request.

## Abbreviations

Cq: Quantification cycle
EGFP: Enhanced green fluorescent protein
ITS1: Internal transcribed spacer-1
MP: Maximum parsimony
rDNA: Ribosomal DNA
