# Supplementary material for: Molecular analysis suggests that Namibian cheetahs (Acinonyx jubatus) are definitive hosts of a so far undescribed Besnoitia species
Source: Parasit Vectors. 2021 Apr 14;14:201. doi: 10.1186/s13071-021-04697-3 (PMC8048190; doi:10.1186/s13071-021-04697-3)
Supplement: Supplementary file 1 — Additional file 1: Table S1. Primer sequences used to amplify rDNA in two aliquots of a faecal sample of a Namibian cheetah with the aim to characterise an unknown Besnoitia sp. [file 13071_2021_4697_MOESM1_ESM.docx]

**Additional file 1: Table S1.**

Primer sequences used to amplify rDNA in two aliquots of a faecal sample of a Namibian cheetah with the aim to characterise an unknown *Besnoitia* sp.

| **Primer designation** | **Sequence of primers 5’-3’** | **Reference*** |
| --- | --- | --- |
| COC-1 | AAG TAT AAG CTT TTA TAC GGC T | [1] |
| COC-2 | CAC TGC CAC GGT AGT CCA ATAC | [1] |
| JS4 | CGA AAT GGG AAG TTT TGT GAA | [2] |
| TIM11 | CAC TGA AAC AGA CGT ACC | [3] |
| BdanjoRev | CAC CAT ACT TCC CGA ATG CAC | [4] |
| BbGS1F | TCG GCG ACG GAT CAT TCA AGT | [5] |
| BbGS1R | ATG CCC CCA ACC GTC CCT ATT A | [5] |
| BbGS2F | GGA TTT CGG CCC TAT TTT G | [5] |
| BbGS2R | CGC GTG CAG CCC AGA ACA | [5] |
| BbGS3F | TGA CGG AAG GGC ACC ACC AG | [5] |
| BbGS3R | TCA CCG GAA CAC TCA ATC | [5] |
| BbGS4F | GAC TCA ACA CGG GGA AAC TCA | [5] |
| BbGS4R | TCA CCT ACG GAA ACC TT | [5] |
| BbGS5F | CTG GTA GCG CTT CAC ACT TCA TTG | [5] |
| BbGS5R | GTT TCA GCC TTG CGA CCA TAC TCC | [5] |
| BbGS6F | CTG CCA GTA GTC ATA TGC TTG TCT | [5] |
| BbGS6R | GCG CCT GCT GCC TTC CTT AG | [5] |
| 18S-5F | CTT AAC CTG CTA AAT AGG ATC AGG | This study |

*Reference

1. Ho MSY, Barr BC, Marsh AE, Anderson ML, Rowe JD, Tarantal AF, et al. Identification of bovine *Neospora* parasites by PCR amplification and specific small-subunit rRNA sequence probe hybridization. Journal of Clinical Microbiology. 1996;34:1203-8.

2. Slapeta JR, Koudela B, Votypka J, Modry D, Horejs R, Lukes J. Coprodiagnosis of *Hammondia heydorni* in dogs by PCR based amplification of ITS 1 rRNA: differentiation from morphologically indistinguishable oocysts of *Neospora caninum*. Vet J. 2002;163 2:147-54.

3. Payne S, Ellis J. Detection of *Neospora caninum* DNA by the polymerase chain reaction. International Journal for Parasitology. 1996;26:347-51.

4. Schares G, Dubey JP, Rosenthal B, Tuschy M, Bärwald A, Conraths FJ. Sensitive, quantitative detection of *Besnoitia darlingi* and related parasites in intermediate hosts and to assess felids as definitive hosts for known and as-yet undescribed related parasite species. International journal for parasitology Parasites and wildlife. 2020;11:114-9.

5. Schares G, Basso W, Majzoub M, Cortes HC, Rostaher A, Selmair J, et al. First in vitro isolation of *Besnoitia besnoiti* from chronically infected cattle in Germany. Vet Parasitol. 2009;163 4:315-22.
