# Supplementary material for: Molecular analysis suggests that Namibian cheetahs (Acinonyx jubatus) are definitive hosts of a so far undescribed Besnoitia species
Source: Parasit Vectors. 2021 Apr 14;14:201. doi: 10.1186/s13071-021-04697-3 (PMC8048190; doi:10.1186/s13071-021-04697-3)
Supplement: Supplementary file 2 — Additional file 2: Text S1. Sequence alignment to assemble the ITS1 rDNA and part of the 18S and 5.8S rDNAs of a B. darlingi-like parasite (here called “Besnoitia-acinonyx”) of Namibian cheetah (Acinonyx jubatus). The text file shows 12 amplicon-based sequences used to establish MW468050. [file 13071_2021_4697_MOESM2_ESM.docx]

**Additional file 2: Text S1.**

Sequence alignment to assemble the ITS1 rDNA and part of the *18S* and *5.8S* rDNAs of a *B. darlingi*-like parasite (here called “Besnoitia-acinonyx”) of Namibian cheetah (*Acinonyx jubatus*). The text file shows 12 amplicon-based sequences used to establish GenBank sequence MW468050.

MT_09_20_1-------------------------------------------------------------------------------------------------------------------------------------------------------------------------------------------------------------------------------------------------------------------------------GCTGCACGCGCGCTACACTGATGCATCCNAACGAGTTTATAACCTTGGCCGATATGTCTAGGTAATCTTGTGAGTATGCATCGTGATGGGGATAGATTATTGCAATTATTAATCTTCAACGAGGAATGCCTAGTAGGCGCAAGTCAGCAGCTTGCGCCGATTACGTCCCTGCCCTTTGTACACACCGCCCGTCGCTCCTACCGATTGAGTGTTCCGGTGAATTATTCGGACCGTTTTGTGGCGCGTTTGTGCCCGAAATGGGAAGTTTTGTGAACCTTAACACTTAGAGGAAGGAGAAGTCGTAACAAGGTTTCCGTAGGTGAACCTGCGGAAGGATCATTCACACGTTGTCCTTTTGACATTTTAACCATTCAACCTTTGAACCCCCCTATTCATCAATGAGCTTGCATCTCTCGTTTCGAGGGGTGCATTCGGGAAGTATGGTGTTCCTTTGTTGTCTTTTTGACAACAAAGCTATCCACCTTCTTCCCTTTTGTTTTTCCCAACACCGTTGACAAAAATCAGAGAGTCTGTTGATTAGCAAGTGAGGGGATTTCGTCCTCTGGGCTCTGCAATCACAGATTCATTAACAAAAGAGTTTTGTATTTTAAATTTTCAGCAATGGATGTCTTGGCTCGCGCAACGATGAAGGACGCAGCGAAATGCGAAACGCAATGTGAATTGCAGAANTTCAGTGAATCATCAGATTCCTGAACGCAAATGGCACCTTN--------------------------------

MT_09_20_1------------------------------------------------------------------------------------------------------------------------------------------------------------------------------------------------------------------------------------------------------------------------------NGCTGCACGCGCGCTACACTGATGCATCC-AACGAGTTTATAACCTTGGCCGATAGGTCTAGGTAATCTTGTGAGTATGCATCGTGATGGGGATAGATTATTGCAATTATTAATCTTCAACGAGGAATGCCTAGTAGGCGCAAGTCAGCAGCTTGCGCCGATTACGTCCCTGCCCTTTGTACACACCGCCCGTCGCTCCTACCGATTGAGTGTTCCGGTGAATTATTCGGACCGTTCTGTGGCGCGTTTGTGCCCGAAATGGGAAGTTTTGTGAACCTTAACACTTAGAGGAAGGAGAAGTCGTAACAAGGTTTCCGTAGGTGAACCTGCGGAAGGATCATTCACACGTTGTCCTTTTGACATTTTAACCATTCAACCTTTGAACCCCCCTATTCAACAATGAGCTTGCATCTCTCGTTTCGAGGGGTGCATTCGGGAAGTATGGTGTTCCTTTGTTGTCTTTTTGACAACAAAGCTATCCGCCTTCTTCCCTTTTGTTTTTCCCAACACCGTTGACAAAAATCAGAGAGTCTGTTGATTAGCAAGTGAGGGGATTTCGTCCTCTGGGCTCTGCAATCACAGATTCATTAACAAAAGAGTTTTGTATTTTAAATTTTCAGCAATGGATGTCTTGGCTCGCGCAACGATGAAGGACGCAGCGAAATGCGAAACGCAATGTGAATTGCAGAA-CTCAGCGAATCATCAGATTTCTGAACGCAAATGGCACCTTGG-------------------------------

MT_09_20_1----------------------------------------------------------------------------------------------------------------------------------------------------------------------------------------------------------------------------------------------------------GATGCCMTTAGATGTTCTGGNGCTGCACGCGCGCTACACTGATGCATCC-AACGAGTTTATAACCTTGGCCGATAGGTCTAGGTAATCTTGTGAGTATGCATCGTGATGGGGATAGATTATTGCAATTATTAATCTTCAACGAGGAATGCCTAGTAGGCGCAAGTCAGCAGCTTGCGCCGATTACGTCCCTGCCCTTTGTACACACCGCCCGTCGCTCCTACCGATTGAGTGTTCCGGTGAATTATTCGGACCGTTTTGTGGCGCGTTTGTGCCCGAAATGGGAAGTTTTGTGAACCTTAACACTTAGAGGAAGGAGAAGTCGTAACAAGGTTTCCGTAGGTGAACCTGCGGAAGGATCATTCACACGTTGTCCTTTTGACATTTTAACCATTCAACCTTTGAACCCCCCTATTCAACAATGAGCTTGCATCTCTCGTTTCGAGGGGTGCATTCGGGAAGTATGGTGTTCCTTTGTTGTCTTTTTGACAACAAAGCTATCCACCTTCTTCCCTTTTGTTTTTCCCAACACCGTTGACAAAAATCAGAGAGTCTGTTGATTAGCAAGTGAGGGGATTTCGTCCTCTGGGCTCTGCAATCACAGATTCATTAACAAAAGAGTTTTGTATTTTAAATTTTCAGCAATGGATGTCTTGGCTCGCGCAACGATGAAGGACGCAGCGAAATGCGAAACGCAATGTGAATTGCAGAANTTCAGTGAATCATCAGATTTCTGAACGCAAATGGCACCTTGGG------------------------------

GS4_Bdar_3TTGACTCAACACGGGGAAACTCACCAGGTCCAGACATAGGAAGGATTGGCAGATTGATAGCTCTTTCTTGATTCTATGGGTGGTGGTGCATGGCCGTTCTTAGTTGGTGGAGTGATTTGTCTGGTTAATTCCGTTAACGAACGAGACCTTAACCTGCTAAATAGGATCAGGAACTTCGTGTTCTTGTATCACTTCTTAGAGGGACTTTGCGTGTCTAACGCAAGGAAGTTTGAGGCAATAACAGGTCTGTGATGCCCTTAGATGTTCTGG-GCTGCACGCGCGCTACACTGATGCATCC-AACGAGTTTATAACCTTGGCCGATAGGTCTAGGTAATCTTGTGAGTATGCATCGTGATGGGGATAGATTATTGCAATTATTAATCTTCAACGAGGAATGCCTAGTAGGCGCAAGTCAGCAGCTTGCGCCGATTACGTCCCTGCCCTTTGTACACACCGCCCGTCGCTCCTACCGATTGAGTGTTCCGGTGAATTATTCGGACCGTTTTGTGGCGCGTTTGTGCCCGAAATGGGAAGTTTTGTGAACCTTAACACTTAGAGGAAGGAGAAGTCGTAACAAGGTTTCCGTAGGTGAACCTGCGGAAGGATCATTCACACGTTGTCCTTTTGACATTTTAACCATTCAACCTTTGAACCCCCCTATTCAACAATGAGCTTGCATCTCTCGTTTCGAGGGGTGCATTCGGGAA-------------------------------------------------------------------------------------------------------------------------------------------------------------------------------------------------------------------------------------------------------------------------------------------------------------------------------------

GS4_Bdar_2--------------------------------------------------------ATAGCTCTTTCTTGGTTCTATGGGTGGTGGTGCATGGCCGTTCTTAGTTGGTGGAGTCATTTGTCTGGTTAATTCCGTTAACGAACGAGACCTTAACCTGCTAAATAGGATCAGGAACTTCGTGTTCTTGTATCACTTCTTAGAGGGACTTTGCGTGTCTAACGCAAGGAAGTTTGAGGCAATAACAGGTCTGTGATGCCCTTAGATGTTCTGG-GCTGCACGCGCGCTACACTGATGCATCC-AACGAGTTTATAACCTTGGCCGATAGGTCTAGGTAATCTTGTGAGTATGCATCGTGATGGGGATAGATTATTGCAATTATTAATCTTCAACGAGGAATGCCTAGTAGGCGCAAGTCAGCAGCTTGCGCCGATTACGTCCCTGTCCTTTGTACACACCGCCCGTCGCTCCTACCGATTGAGTGTTCCGGTGAATTATTCGGACCGTTTTGTGGCGCGTTTGTGCCCGAAATGGGAAGTTTTGTGAACCTTAACACTTAGAGGAAGGAGAAGTCGTAACAAGGTTTCCGTAGGTGAACCTGCGGAAGGATCATTCACACGTTGTCCTTTTGACATTTTAACCATTCAACCTTTGAACCCCCCTATTCAACAATGAGCTTGCATCTCTCGTTTCGAGGGGTGCATTCGGGA--------------------------------------------------------------------------------------------------------------------------------------------------------------------------------------------------------------------------------------------------------------------------------------------------------------------------------------

GS4_Bdar_1TTGACTCAACACGGGGAAACTCACCAGGTCCAGACATAGGAAGGATTGACAGATTGATAGCTCTTTCTTGATTCTATGGGTGGTGGTGCATGGCCGTTCTTAGCTGGCGGAGTGATTTGTCTGGTTAATTCCGTTAACGAACGAGACCTCAACCTGCTAAATAGGATCAGGAACTTCGTGTTCTTGTATCACTTCTTAGAGGGACTTTGCGTGTCTAACGCAAGGAAGTTTGAGGCAATAACAGGTCTGTGATGCCCTTAGATGTTCTGG-GCTGCACGCGCGCTACACTGATGCATCC-AACGAGTTTATAACCTTGGCCGATAGGTCTAGGTAGTCTTGTGAGTATGCATCGTGATGGGGATAGATTATTGCAATTATTAATCTTCAACGAGGAATGCCTAGTAGGCGCAAGTCAGCAGCTTGCGCCGATTACGTCCCTGCCCTTTGTACACACCGCCCGTCGCTCCTACCGATTGAGTGTTCCGGTGAATTATTCGGACCGTTTTGTGGCGCGTTTGTGCCCGAAATGGGAAGTTTTGTGAACCTTAACACTTAGAGGAAGGAGAAGTCGTAACAAGGTTTCCGTAGGTGAACCTGCGGAAGGATCATTCACACGTTGTCCTTTTGACATTTTAACCATTCAACCTTTGAACCCCCCTATTCAACAATGAGCTTGCATCTCTCGTTTCGAGGGGTGCATTCG-----------------------------------------------------------------------------------------------------------------------------------------------------------------------------------------------------------------------------------------------------------------------------------------------------------------------------------------

JS4_TIM11_----------------------------------------------------------------------------------------------------------------------------------------------------------------------------------------------------------------------------------------------------------------------------------------------------------------------------------------------------------------------------------------------------------------------------------------------------------------------------------------------------------------------------------------------------------------------------------------------------------------GAACCTGCGGAAGGATCATTCACACGTTGTCCTTTTGACATTTTAACCATTCAACCTTTGAACCCCCCTATTCAACAATGAGCTTGCATCTCTCGTTTCGAGGGGTGCATTCGGGAAGTATGGTGTTCCTTTGTTGTCTTTTTGACAACAAAGCTATCCRCCTTCTTCCCTTTTGTTTTTCCCAACACCGTTGACAAAAATCAGAGAGTCTGTTGATTAGCAAGTGAGGGGATTTCGTCCTCTGGGCTCTGCAATCACAGATTCATTAACAAAAGAGTTTTGTATTTTAAATTTTCAGCAATGGATGTCTTGGCTCGCGCAACGATGAAGGACGCAGCGAAATGCGAAACGCAATGTGAATTGCAGAA-TTCAGTGAATCATCAGATTTCTGAACG----------------------------------------------

MT_09_20_1-----------------------------------------------------------------------------------------------------------------------------------------------------------------------------------------------------------------------------------------------------------------------------------------------------------------------------------------------------------------------------------------------------------------------------------------------------------------------------------------------------------------------------------------------------------------------------------------GTCGTAACAAGGTTTCCGTAGGTGAACCTGCGGAAGGATCATTCACACGTTGTCCTTTTGACATTTTAACCATTCAACCTTTGAACCCCCCTATTCAACAATGAGCTTGCATCTCTCGTTTCGAGGGGTGCATTCGGGAAGTATGGTGTTCCTTTGTTGTCTTTTTGACAACAAAGCTATCCACCTTCTTCCCTTTTGTTTTTCCCTACACCGTTGACAAAAATCAGAGAGTCTGTTGATTAGCAAGTGAGGGGATTTCGTCCTCTGGGTTCTGCAATCACAGATTCATTAACAAAAGAGTTTTGTATTTTAAATTTTCAGCAATGGATGTCTTGGCTCGCGCAACGATGAAGGACGCAGCGAAATGCGAAACGCAATGTGAATTGCAGAA-TTCAGTGAATCATCAGATTTCTGAACGCAAATGGCACCTTGGGGATACTCTCCTTGGTACGTCTGTTTCAGTG

MT_09_20_1-----------------------------------------------------------------------------------------------------------------------------------------------------------------------------------------------------------------------------------------------------------------------------------------------------------------------------------------------------------------------------------------------------------------------------------------------------------------------------------------------------------------------------------------------------------------------------------------GTCGTAACAAGGTTTCCGTAGGTGAACCTGCGGAAGGATCATTCACACGTTGTCCTTTTGACA-TTTAACCATTCAACCTTTGAACCCCCCTATTCAACAATGGGCTTGCATCTCTCGTTTCGAGGGGTGCATTCGGGAAGTATGGTGTTCCTTTGTTGTCCTTTTGACAACAAAGCTATCCACCTTCTTCCCTTTTGTTTTTCCCAACACCGTTGACAAAAATCAGAGAGTCTGTTGATTAGCAAGTGAGGGGATTTCGTCCTCTGGGCTCTGCAATCACAGATTCATTAACAAAAGAGTTTTGTATTTTAAATTTTCAGCAATGGATGTCTTGGCTCGCGCAACCATGAAGGACGCAGCGAAATGCGAAACGCAATGTGAATTGCAGAA-TTCAGTGAATCATCAGATTTCTGAACGCAAATGGCACCTTGGGGATACTCTCCTTGGTACGTCTGTTTCAGTG

JS4_TIM11_------------------------------------------------------------------------------------------------------------------------------------------------------------------------------------------------------------------------------------------------------------------------------------------------------------------------------------------------------------------------------------------------------------------------------------------------------------------------------------------------------------------------------------------------------------------CTTAACACTTAGAGGAAGGAGAAGTCGTAACAAGGTTTCCGTAGGTGAACCTGCGGAAGGATCATTCACACGTTGTCCTTTTGACATTTTAACCATTCAACCTTTGAACCCCCCTATTCAACAATGAGCTTGCATCTCTCGTTTCGAGGGGTGCATTCGGGAAGTATGGTGTTCCTTTGTTGTCTTTTTGACAACAAAGCTATCCACCTTCTTCCCTTTTGTTTTTCCCAACACCGTTGACAAAAATCAGAGAGTCTGTTGATTAGCAAGTGAGGGGATTTCGTCCTCTGGGCTCTGCAATCACAGATTCATTAACAAAAGAGTTTTGTATTTTAAATTTTCAGCAATGGATGTCTTGGCTCGCGCAACGATGAAGGACGCAGCGAAATGCGAAACGCAATGTGAATTGCAGAA-TTCAGTGAATCATCAGATTTCTGAACGCAAATGGCACCTTGGGGATACTCTCCTTGGTACGTCTGTTTCAGT-

JS4_TIM11_----------------------------------------------------------------------------------------------------------------------------------------------------------------------------------------------------------------------------------------------------------------------------------------------------------------------------------------------------------------------------------------------------------------------------------------------------------------------------------------------------------------------------------------------------------------------------------------------------------------------------GGATCATTCACACGTTGTCCTTTTGACATTTTAACCATTCAACCTTTGAACCCCCCTATTCAACAATGAGCTTGCATCTCTCGTTTCGAGGGGTGCATTCGGGAAGTATGGTGTTCCTTTGTTGTCTTTTTGACAACAAAGCTATCCACCTTCTTCCCTTTTGTTTTTCCCAACACCGTTGACAAAAATCAGAGAGTCTGTTGATTAGCAAGTGAGGGGATTTCGTCCTCTGGGCTCTGCAATCACAGATTCATTAACAAAAGAGTTTTGTATTTTAAATTTTCAGCAATGGATGTCTTGGCTCGCGCAACGATGAAGGACGCAGCGAAATGCGAAACGCAATGTGAATTGCAGAA-TTCAGTGAATCATCAGATTTCTGAACGCAAATGGCACCTTGGGGATACTCTCCTTGGTACGTCTGTTTCAGTG

JS4_TIM11_------------------------------------------------------------------------------------------------------------------------------------------------------------------------------------------------------------------------------------------------------------------------------------------------------------------------------------------------------------------------------------------------------------------------------------------------------------------------------------------------------------------------------------------------------------------CTTAACACTTAGAGGAAGGAGAAGTCGTAACAAGGTTTCCGTAGGTGAACCTGCGGAAGGATCATTCACACGTTGTCCTTTTGACATTTTAACCATTCAACCTTTGAACCCCCCTATTCAACAATGAGCTTGCATCTCTCGTTTCGAGGGGTGCATTCGGGAAGTATGGTGTTCCTTTGTTGTCTTTTTGACAACAAAGCTATCCACCTTCTTCCCTTTTGTTTTTCCCAACACCGTTGACAAAAATCAGAGAGTCTGTTGATTAGCAAGTGAGGGGATTTCGTCCTCTGGGCTCTGCAATCACAGATTCATTAACAAAAGAGTTTTGTATTTTAAATTTTCAGCAATGGATGTCTTGGCTCGCGCAACGATGAAGGACGCAGCGAAATGCGAAACGCAATGTGAATTGCAGAA-TTCAGTGAATCATCAGATTTCTGAACGCAAATGGCACCTTGGGGATACTCTCCTTGGTACGTCTGTTTCAGTG
